# Parents’ Experience of Having an Infant in the Neonatal Intensive Care Unit: A Qualitative Study

**DOI:** 10.7759/cureus.16747

**Published:** 2021-07-30

**Authors:** Shahd H Rihan, Laila M Mohamadeen, Sara A Zayadneh, Furqan M Hilal, Hidaia A Rashid, Neveen M Azzam, Dua'a J Khalaf, Eman F Badran, Reema R Safadi

**Affiliations:** 1 School of Medicine, The University of Jordan, Amman, JOR; 2 Department of Pediatrics, Neonatal Division, School of Medicine, The University of Jordan, Amman, JOR; 3 Maternal and Child Health Department, School of Nursing, The University of Jordan, Amman, JOR

**Keywords:** hospitalization in nicu, coping, experience, jordan, qualitative research

## Abstract

Introduction: Admission to the neonatal intensive care unit (NICU) is usually unexpected and can be stressful to the parents causing strenuous psychosocial effects. Parents of these infants are subject to suffering stress, depression, and feelings of powerlessness. This study aimed at describing parents’ experience of having their infant in the neonatal intensive care unit.

Method: A qualitative descriptive design was used. Parents (six couples and four mothers) of infants hospitalized for at least ten days regardless of gestational age, gender, or medical diagnosis were selected from a teaching hospital in Amman, Jordan. Semi-structured interviews were conducted between June 2019 and November 2019.

Results: Thematic analysis of the data revealed four emerging themes: (1) Living the ambiguities of the admission to the NICU, (2) Living the burdens of their infants’ hospitalization, (3) Coping with the stresses of a hospitalized infant, and (4) Reflecting on interactions with healthcare staff and the environment.

Discussion and conclusion: The study findings demonstrated parents’ worries and needs and highlighted the use of spirituality/religiosity as a coping mechanism. The findings will guide healthcare providers and policymakers to develop caring strategies that enhance care delivered to parents of infants in intensive care units.

## Introduction

The physical and emotional closeness between parents and their infants is essential for relationship establishment and bonding [1]. Sometimes, a newborn needs to be admitted to the neonatal intensive care unit (NICU) due to certain issues, such as prematurity with respiratory problems, neonatal sepsis, meconium aspiration syndrome, and hyperbilirubinemia [2]. NICU admission is usually unexpected and can be stressful to the parents causing strenuous psychosocial effects [3]. Parents of infants admitted to the NICU often reported suffering stress, depression, and feelings of powerlessness [4].

Heidari et al. described parents’ stress as an experience that carries a great sense of misgiving, nervousness, emotional tension, and separation anxiety [5]. Moreover, Obeidat et al. documented that some mothers experienced emotional instability, guilt, exhaustion, and depression [6]. A study conducted in Jordan reported that all mothers and fathers expressed feelings such as shock, anxiety, and worry when they were informed about their neonate’s admission to the NICU [7]. Parent­-infant separation due to hospitalization was another cause of parental stress [5]. Improvement of the infant’s health created feelings of hopefulness, while the opposite of this resulted in despair [7].

Parents of infants in the NICU have to address multiple challenges. These include handling their premature babies, dealing with people and medical staff in the NICU, and handling their own relationships [8]. Parents’ appetite and sleep patterns were also affected [5]. It is common for parents to have disrupted lifestyles at home due to stressful conditions [7]. Studies found changes such as skipping days at work for the sake of finding peace or having time for visits to the NICU [7]. Additionally, parents’ relationships as spouses were affected as mothers felt isolated from their husbands [6]. But generally, parents were supportive of each other [8].

A study by Heidari et al. found that Iranian parents were concerned about their infant's appearance, their own role as parents, and the worsening of their infant’s health, and death [5]. In another study, it was found that some parents were concerned about the limited information they receive about their infants [9]. Although parents had a great desire to care for their babies in the NICU, they were hesitant about that in fear of not complying with everything they were told [10].

Several studies were conducted to examine the impact of the healthcare team in providing support. Heidari et al. concluded that offering information to parents and engaging them in decision-making regarding their infant, helped in alleviating the anxiety of most parents [5]. This was supported by Hesham et al. who did an interventional study and found that most fathers changed their feelings towards the admission of their neonates to the NICU after the education and the information they received [11]. Additionally, parents' trust in the medical staff has increased when they were provided with knowledge about their baby's condition [10]. In another study, Abuidhail et al. compared parents' experience regarding their infant’s admission to private versus teaching hospitals and found that parents receive more support from nurses at private hospitals than at teaching hospitals [7]. On the other hand, some parents were displeased with their doctor’s attitudes and considered him/her as a source of pain and anger because they did not talk to them [8].

Parents of infants admitted to NICU used different coping mechanisms to enhance their coping. One way to cope with the stress of hospitalization was to compare the current experience with a previous NICU hospitalization [10]. Another way of coping was by finding support through religious faith [6]. Obeidat et al. reported that when parents were encouraged to be engaged in their infant’s care they felt safer, took control of the situation, had more confidence, and felt more connected and related to their infant [4]. Moreover, fathers used different coping strategies from mothers as they increasingly had more needs to normalize their lives [9].

The review of literature has revealed that parents of infants in NICU are affected tremendously as their daily life has changed, and their family relationships are disturbed. To conclude, despite the global literature about parents' concerns regarding their infant’s hospitalization, little is known about Jordanian parents and how they are affected when their infants stay in NICU for at least ten days. Therefore, this study aims to understand parents’ concerns, coping mechanisms, changes in everyday lifestyle, and evaluation of the interactions with healthcare providers. An understanding of this phenomenon in a Jordanian-Arab socio-cultural context that is distinct from western cultures because of its extended family ties and support, distribution of gender roles, and spiritual and religious coping mechanisms is crucial. A thorough understanding of parents’ experience is needed to enhance relevant healthcare services provided to parents and their infants in the NICU.

Research questions

The central research question is: What is the parents’ experience of having their infant hospitalized for at least ten days in the NICU at a teaching hospital in Jordan?

Sub Questions

What are the parents’ feelings and concerns as they learn about the admission of their infant to the NICU?

How does hospitalization affect parents’ daily lifestyle and marital relations?

What kind of support do parents receive as they deal with their infant’s hospitalization?

How do parents cope with the stresses of an infant’s hospitalization to the NICU?

What are the parents’ evaluations of the interactions with health care providers as their infant stays in the NICU?

## Materials and methods

Research design

A qualitative descriptive design was used to understand the experience of Jordanian parents as their infant was hospitalized in the NICU for a prolonged period (≥ ten days). Bradshaw et al. described qualitative research as an approach that seeks to explore and understand a phenomenon, a process, or the perspectives and worldviews of the people involved [12]. It is a method to provide a rich description of the experience depicted in easy and simple language [12].

Setting

The study took place at the neonatal intensive care unit of a teaching hospital in Amman. This hospital handles around 1000 neonatal admissions of different neonatal illnesses and 4000 deliveries per year. The staff in this unit includes two attending and 34 pediatric residents. The pediatric resident team's clinical rotation changes monthly. One clinical pharmacist is routinely available five days per week. Nurse to patient ratio is one: four.

Recruitment

Participants were recruited between June 2019 and November 2019. They were selected with the help of the health staff at NICU according to the eligibility criteria. We contacted each couple individually as they visited the NICU.

Participants 

Parents (Couples or single parents) of infants admitted for at least ten days to NICU at a teaching hospital were targeted. The inclusion criteria were willing parents who had an infant hospitalized to the NICU for at least ten days after birth regardless of the gestational age and gender. 

Ethical consideration

This research was conducted after the approval of the Institutional Review Board (IRB) at Jordan University Hospital (approval number 184/2019 and 269/2019) and Deanship of Scientific Research (approval number 547/2019/19). The participants were given detailed information about the research purpose, procedures, rights to voluntary participation, and the ability to withdraw or to decline participation without penalty. They were assured of privacy and confidentiality of the data. Permission to tape-record the interview was taken before the beginning of the interview. After that, the informed consent was signed and a copy was given to the participant. Each interview was held in a closed room to ensure the privacy of the participants. The tape recordings were anonymized and transcribed. All recordings and transcripts were kept in a safe place where no one other than the researchers can have access. 

Data collection

Data were collected using semi-structured interviews that were developed by the researchers after referring to the literature and textbooks for writing questions [13]. After obtaining participants’ consent, an interview was begun with an open-ended question and this was followed by more probing questions as shown in the interview guide in Table 1.

**Table 1:** Interview guide

	Questions
Open-ended question to be started with:	Tell us about your experience of having your infant hospitalized in the NICU?
Secondary questions to be followed:	Describe your feelings as you learned about the admission to the NICU? What about your husband's feelings? How did hospitalization affect your daily lifestyle and marital relations? How did you cope with the stresses of infant’s hospitalization to the NICU? How did you evaluate the interactions with health care providers while your infant stayed in the NICU?
Probing question to be used when indicated:	Can you clarify it more? Explain how or why.

Interviews lasted between 25-33 minutes and were audio-recorded and transcribed verbatim by the researchers. Data were collected until saturation was achieved on the ninth interview. One more interview was conducted to confirm our decision to stop data collection as no new data were obtained.

Data analysis

Data analysis using thematic analysis was conducted simultaneously with data collection and in an iterative way. Researchers listened to the tape-recorded interviews several times to extract a deeper meaning of concepts. The recorded interviews were transcribed verbatim in the Arabic language to maintain the actual meaning of the verse. Coding was conducted by searching for key meaning units, giving these units a specific code, and highlighting the key meanings across the data set. Next, we developed codes into categories, subthemes, and themes based on the similarities and differences and combined for interrelations and meanings. Intercoder agreement among researchers was established by reviewing and agreeing on meaning units, codes, categories, and themes. Data trustworthiness was established by prolonged engagement with participants in NICU, following specific procedural steps such as a thorough and repetitive reading of raw data for a deeper understanding and assessing the supplemental comments from researchers to correlate and reach consensus to approve extracted codes and categories.

## Results

The study participants included six couples and four mothers due to fathers' engagement at work during the time of the interview. Mothers' age ranged between 20-45 years old, while fathers' age ranged between 27-48 years old. The educational background of the participants varied between the secondary level (n=7), high school (n=1), diploma (n=5), and bachelor's degree (n=3). Seven of the mothers were housewives and the remaining three were employed. Eight of the mothers were multiparous and two were primiparous.

Infants hospitalized in NICU had the following diagnoses: congenital heart disease (n=2), VACTERL Syndrome (vertebral defects, anal atresia, cardiac defects, tracheoesophageal fistula, renal anomalies, and limb abnormalities) (n=1), intestinal obstruction due to congenital hypothyroidism (n=1), duodenal obstruction (n=1), prematurity and associated conditions (n=4), and severe respiratory distress (n=1). All infants were products of normal conception without the assistance of reproductive techniques. There were eight singleton infants and two multi-gestational twins. Only one of the twin gestations was completed until full-term (see Table 2 for demographic data).

**Table 2:** Demographic data of the participants (n=10)

Maternal data	
Age	Mean = 30.4, Range (20-45)
Religion (Islam)	100%
Education	Secondary level (n=1) High school (n= 3) Diploma (n=3) Bachelor's degree (n=3)
Occupation	Housewife (n=7) Teacher (n=2) Nurse (n=1)
Paternal data	
Age	Mean = 37.5, Range (27-48)
Religion (Islam)	100%
Education	Secondary level (n=4) High school (n= 2) Diploma (n=2) Bachelor's degree (n=2)
Occupation	Doctor (n=1) Free job (n=1) Designer (n=1) Restaurant worker (n=1) Employee (n= 3) Teacher (n=1) Imam of a mosque (n=1) Military/ Soldier (n=1)
Family data	
Income per month	Mean= 519.8, Range (118- 900)
Have health insurance	100%
Number of children in the family (including this infant)	Mean=3, Range (1-7)
Previous NICU admission of his/her siblings	No (n=8), Yes (n=2)
Duration of admission of the sibling	1 hour (n=1), 2 day (n=1)
Reason for admission of the sibling	Respiratory distress (n=1), Jaundice (n=1)
Infant data	
Conceiving way (Normal or using assisted reproductive techniques)	100% normal
Age of infant on interviewing parents	Mean = 20.6 day, Range (10- 42 day)
Number of fetuses during pregnancy	One (n=8), twin (n=2)
Order in the family	Mean = 3, Range (1-7)
Duration of admission until the date of interview	Mean=19.5 day, Range (10-42 day)
Reason for admission	Congenital anomaly (n=5) Prematurity and associated conditions (n=4) Severe respiratory distress (n=1).

The study findings are summarized into four main themes: (1) Living the ambiguities of admission to the NICU, (2) Living the burdens of their infants’ hospitalization, (3) Coping with the stresses of a hospitalized infant, and (4) Reflecting on interactions with healthcare staff and the environment.

Theme-1: Living the ambiguities of admission to the NICU

As the infant was admitted to the NICU, parents’ worries about the admission and its consequences have emerged. There was an immediate response to the why’s and how’s to manage the adversity of the situation, and worries about the present moment and the future. These responses are captured in the following two subthemes.

Subtheme-1: Reacting to the News: Uncertainties About Why and How to Manage Care

This subtheme includes the parental feelings when they heard the news, searching for the cause of the admission, longing for discharge, and lack of self-confidence.

All mothers and most fathers expressed being worried, anxious, shocked, and stressed when they first heard of their infants' admission to the NICU. They described their experience as ‘difficult’ that needed to cope with. They felt separated from their infant and cried for that reason. One mother said: “I keep crying all day long at home. I want to be near my baby.” (Mother-10). Moreover, most mothers and some fathers shared feelings of sorrow for their babies. One mother complained about the difficulty of the situation but tried to console herself by saying: “one must be practical and think logically. I mean, I cannot push for an early discharge of my baby. After all, he is there just for weight gain and nothing serious like needing oxygen or whatever. It is for his benefit.” (Mother-6)

All parents described their longing to know the cause of the admission. They asked questions such as: “what happened?”. Their expressions were manifested by confusion, seeking knowledge, or feeling guilty by blaming themselves for having a preterm infant.

“Everybody told me to take it easy and take enough rest, it is my fault, I did not listen.” (Mother-7)

“Every time I sit down I start thinking of the baby. I cannot sleep because of my continuous thinking.” (Father-4)

Most mothers were excited and happy about having their babies discharged from the NICU. For example, one mother said:

“Her discharge became our only job. When is she to be out? I wish it happens now, with me now.” (Mother-6)

Alternatively, only one mother, whose infant was diagnosed with VACTERL Syndrome, had mixed feelings. She was worried about how to take care of her infant when taken home. This mother felt that the hospital environment was a better-suited place for her infant. This mixed feeling was expressed by mother-4 who said: “No matter how much they take care of him; it is not like when he is with his mom. Nevertheless, he needs special care which I cannot offer him at home.” (Mother-4)

Subtheme-2: Present and Future Worries About the Baby

This subtheme describes the parents' worries about their infants in NICU. This includes concerns about their infants’ diagnosis, their future, leaving them in the NICU, reaction to the medical devices, financial burden, and unexpected longer hospitalization. Most mothers shared the same concerns about leaving their infant in the NICU, as well as worrying about longer hospitalization.

“I worry, and wonder about what is happening to her. I am afraid because she has stayed so long in the NICU !!” (Mother-2)

“When I go home after visiting the NICU I keep thinking about my baby. I like to stay with him all the time. Sometimes, I feel that there, (in NICU) he is at more risk.” (Mother-9)

Most parents were concerned about losing their infant and about their future. A mother of a preterm infant stated: “The most important thing to me is that my baby has no medical problem; this has comforted me.” (Mother-5). But mothers of infants with congenital anomalies had more obvious worried expressions, as one stated: “I am worried and sad for him. I am afraid that he will need someone to look after him for the rest of his life. I won’t live forever to look after him, and I am afraid that his siblings must look after him, would they accept him? I cannot enroll him in a special needs school.” (Mother-4).

Parents were also worried about their infants being attached to machines. They were concerned about what would happen to their infant when disconnected from the machines at home. One mother said: “He needs special medical devices which are not available at home. There would be lots of difficulties and stresses for me at home” (Mother-4). Machines' discontinuation was also an indicator of the infant’s improvement. One mother said: “We were very tense until they told us that they would stop the respiratory machines. This was a relief! Now we know that he is in a stable condition.” (Mother-1)

Some parents also expressed the effect of having insurance on relieving their anxiety by saying: “The most important thing is that I recently obtained health insurance. My worries about the NICU expenses are gone now and I feel much better”. (Father-9)

Theme-2: Living the burdens of their infants’ hospitalization

Parents voiced a change in their daily life because of the infant’s hospitalization in NICU. Parents reported their difficulties in three subthemes: 1. Spending time between home and hospital, 2. Breastfeeding issues, and 3. Disruption of the family norms.

Subtheme-1: Spending Time Between Home and Hospital

Most mothers expressed time-wasting because of the frequent need to be in the hospital for breast milk pumping and because of the frequent visits to NICU.

“My life is upside down now. I cannot stay at home anymore. I spend most time in the hospital now.” (Mother-7)

“I come here daily. This is very tiresome as I remain in the hospital.” (Mother-10)

To these mothers, trips to the hospital were difficult because they had to take public transportation to get to the hospital.

“Transportation, as you know, is very difficult here in Amman”. (Mother-5).

The consequences of infants' staying in NICU were also affecting fathers’ work schedules as they had problems at work and they had to miss workdays because of frequent hospital visits.

“I shifted from daytime to nighttime work schedule to fit with hospital visiting hours.” (Father-3)

Subtheme-2: Breastfeeding Issues

All mothers expressed problems with breastfeeding. Some mothers criticized doctors' insistence on providing the infant with breast milk, while others complained about other difficulties including breast milk pumping, delivering this milk to the hospital, and coming to NICU for breastfeeding.

“They ask for large amounts of breast milk which was difficult to supply. Timing was inconsistent and inconvenient to provide.” (Father-1)

“It was very troublesome, I had so much pain in doing this.” (Mother-5)

Subtheme-3: Disruption of the Family Norms

Mothers also complained about neglecting their spouses, home responsibilities, and other children at home.

“I was unable to help my kids with their school work, nor prepare them for the exams. I couldn't look after my house or my husband. I couldn't carry my household responsibilities as I used to.” (Mother-9)

Most parents reported cooperation in the family. Partners and other female family members were helpful to mothers as they offered various kinds of assistance.

“I am here at the NICU and my wife is at home with the other twin son. My family is helping too. They look after our son so that she can do breast milk pumping for the hospitalized son.” (Father-1)

Theme-3: Coping with the stresses of a hospitalized infant

It was clear that parents utilized different ways to cope with this insidious difficulty. The common features of used coping strategies were self-developed and are culturally prevalent; none of the mothers reported a structured formal method for helping them in this situation. Statements expressing spiritual practices, distraction, acquiescence, comparing with previous experiences, and family and friends support were frequently stated as part of the accounts.

One of the most frequent reports referred to reliance on God’s mercy. All parents expressed comfort and peace by seeking God’s help, reading Qur’an, praying, trusting, or thanking God.

“We believe in the wisdom of Allah. I mean, we as Muslims, we believe in the rule of Allah’s will. It is Allah’s wish, our fate, and whatever He decides for us, we have to accept.” (Father-1)

Some parents described distraction to keep their thinking away. They tried to keep engaged by being with others or visiting their infant.

“When I am all alone I keep thinking about my baby; that is why I prefer to be in the company of others. Additionally, I like visiting my baby at the NICU because I feel relieved when I see her.” (Mother-9)

Time was also helpful in accepting the condition.

"We are trying to adjust to it. You feel you can absorb the situation day after day as time goes by.” (Mother-1)

Comparing the situation with a previous experience was another way that made some mothers feel better regarding their infant’s hospitalization in NICU.

“My mum was supportive; she kept telling me that my nephews went through the NICU and made it out, nothing to worry about.” (Mother-7)

All husbands showed support for their wives. They tried hard to ease the suffering by offering possible help. Additionally, in all interviews, mothers and fathers received their family’s support whenever needed.

“They didn't leave me alone, my husband and his family were supportive, my family was supportive. They tried to comfort me and make me feel well.” (Mother-5)

One husband said: “I tried to stay strong in front of my wife and tried to make things look like normal. I gave her the news bit by bit so that she is not shocked. I know, as a woman she is not as strong as a man. She would be devastated to suddenly know the news.” (Father-8)

Theme-4: Reflecting on interactions with healthcare staff and the environment

Most parents praised the level of care provided by the health staff and expressed their satisfaction with the medical-staff cooperation, care, education about care, and communication. Moreover, some parents showed great trust and reliance on the health staff and pointed out their psychological support.

“The nurses treated my son as if he was their own! if you ever had a question, the medical staff would give you all the information you need. They even answered my phone calls! And always kept me informed, you feel relaxed that your baby is with them.” (Mother-10)

“The doctors and residents never turn you down. They are all so kind, all are smiling. I did not expect such an excellent treatment.” (Mother-4)

## Discussion

This study aimed at describing the experience of parents (fathers and mothers) of an infant admitted to the NICU for a prolonged period, at least ten days, in the Jordanian context. The study depicts the cultural and religious background of parents as a context that may have influenced the experience of hospitalization of infants. Parents’ concerns, coping mechanisms, change in everyday lifestyle, and evaluations of the interactions with healthcare providers were explored. We found that parents described their experience as a ‘difficult’ situation with feelings of sorrow for their babies that required appropriate coping. Their reaction to the NICU admission was that of shock, worry, anxiety, and stress. These findings are consistent with a recent study conducted by Abuidhail et al. which found that parents of sick or preterm infants who were hospitalized for at least 24 hours experienced shock, worry, and anxiety [7]. In this study, six mothers with hospitalized infants for a longer period expressed feelings of separation from their infant. This separation from the infant was different for four mothers, three of whom could spend more time with their infants in the NICU and the fourth had one of the twin babies with her at home. This finding is congruent with another study conducted in Norway, which reported that parents found it difficult to bond with their preterm babies when they were separated from them for a long period in the hospital [10].

In reaction to admission to NICU, all parents sought knowledge about the cause of admission. Parents were confused and had many questions about that, especially parents of babies with congenital anomalies. This study reported that some parents blamed themselves for having a preterm infant. This finding is consistent with another Iranian study's findings, which included 21 participants who blamed themselves for the unexpected pregnancy [5].

Admission to the NICU was a source of confusion and anxiety, and discharge from NICU was a relief. Mothers were happy to hear that their infant is discharged despite their inner feeling that NICU care is a better environment for their infant. All mothers had a feeling of relief except for one whose infant was diagnosed with VACTERL Syndrome. She was concerned about after-discharge care and lack of confidence in looking after her infant at home. This finding is inconsistent with Zamanzadeh et al. who found that most parents preferred that their infant stays in NICU until they were fully capable of taking good care of them [14].

Whereas most mothers in this study expressed their concerns about leaving their infant in the NICU and about a prolonged hospitalization, fathers showed less anxiety and perhaps ‘giving in’ to the situation. Similarly, Obeidat et al. reported concern regarding future complications of growth and development due to infant’s hospitalization in NICU [4]. Also, Hagen et al. reported that both parents were uncomfortable as they thought that babies are fragile and only a few steps away from death in NICU [10].

Worrying about the infant’s future and health condition was a salient finding. In this study, we found that all mothers and most fathers were afraid about their infant’s health status and expressed their fears about losing their infant. It was observed that parents of infants with congenital anomalies, especially those with Down Syndrome, were more concerned about the future of their children, and this phenomenon was more prevalent among mothers than fathers. Similarly, researchers in Iran reported that most of the parents behaved differently with varying levels of agitation and anxiety, worries about the infants’ future, as well as constant checking on the infant's health status [5].

Another important concern of parents in this study was about having their infant attached to machines. For some parents, it was perceived as scary and worrying, and the discontinuation of the machines was a relief and a sign of improvement. Similarly, this result is congruent with a Canadian study that found that although mothers' appreciated the advantages of supplemental oxygen delivery, they were worried about the health consequences of these devices [15]. Admission to NICU was also associated with worries about the financial burdens it has on the family. Having or seeking health insurance was a source of relief in this critical time.

The findings of this study show that most parents expressed a change in their daily lifestyle. Parents in this study described difficulties in making NICU visits. Of these problems, mothers complained of transportation and distance issues and fathers complained about missing days at work or changing their time schedules at work to meet the hospital requirements. This result is congruent with Abuidhail et al.'s results which found that Jordanian parents of sick or preterm infants had to change their normal daily routine, skip going to work, and stay at home to pray and visit their infants [7]. 

All mothers in the current study expressed problems with breastfeeding support that varied between criticizing physicians' insistence on breastmilk and complaints about breastfeeding difficulties while their babies were still in NICU. Mothers had to express milk at home and had it carried or sent to the hospital in case they were unable to be in the hospital personally. This result is congruent with Fernández Medina et al. findings which reported difficulties that mothers have to deal with when they provide their own milk to their extremely preterm infants [16]. In this study, mothers complained of neglecting other children at home and ignoring husbands' needs and home responsibilities. This result was also supported by Obeidat et al. who found that mothers felt isolated from their families, failed to meet the family needs, and were focused on their infant in the crisis [6]. Similar to Hagen et al.'s findings, most parents in this study reported getting support from their own families [10]. Partners and other female family members’ engagement offered all sorts of assistance including cooking, attending to children, and house chores.

Parents used several coping mechanisms to adapt to NICU hospitalization difficulties. Adherence to religious beliefs was a major finding. Religious words of comfort were present in every interview. All parents described themselves as relying on God’s help to save their infant. They prayed and recited the holy Quran pleading for His assistance. This finding is consistent with another Jordanian study that reported that parents tried to normalize their lives and strengthen themselves through practicing rituals of spiritual beliefs [6]. Some families in this study expressed their acceptance of the condition because denial will not alleviate their fears. Time was a factor in accepting. With time passing, parents were more conceding with the ailment of their infant.

Distraction was also used to cope with the infant's NICU hospitalization. This study found that maternal socialization with other family members, by expressing and discussing their emotions and by comparing with other family member's previous positive experiences helped them in coping with the situation. Similarly, Smith et al. reported that parents socializing and engaging with other parents in NICU was a helpful strategy to cope [17]. Also, in this study, it was found that parents' visits to their infants in the NICU helped them in coping. Contrastingly, Smith et al. reported that getting away from the NICU was a coping strategy that parents used [17].

Husband’s support was another way of coping as reported by most mothers. In this study, we found that husbands tried hard to ease the suffering of their spouses by offering all possible help and engagement. Additionally, the family’s support to the parents existed whenever needed. Smith et al. verified the importance of family and friends’ engagement in supporting parents, with husbands being the central source of emotional relief and support [17].

In a final theme, parents offered their views regarding their interactions with the healthcare providers. Most parents praised the level of care provided and expressed their satisfaction with healthcare provision and communication. Some parents showed great trust and reliance on the health staff and pointed out their psychological support. This result is congruent with Abuidhail et al. who reported parents’ satisfaction with received care from the healthcare professionals at private or teaching hospitals in Jordan [7].

Limitations

This research was limited to one hospital which limits the transferability of the study. Generalizability is not an aim in qualitative research; however, having limited the experiences to one location does not yield variation of the experience and perspectives of participants. Participants could have given us socially acceptable answers to please us as part of the medical team in the hospital. Therefore, further research studies are needed to understand the needs of parents of infants admitted to the NICU.

## Conclusions

Having an infant in the NICU was a stressful experience associated with many difficulties. Most parents expressed shock, sadness, crying, worries about their infant's health, and the desire to know the cause of admission. This experience affects the whole family in many aspects including daily life changes, time-wasting, father's work problems, breastfeeding difficulties, and less attention to household and children’s responsibilities. Coping mechanisms that helped parents to cope included spirituality, distraction, acceptance, comparing with previous experiences, and family and friends support. The parents in this study were satisfied with the health-staff care and support, and health education given as they cared for their infants.

Implication for practice

The findings of this study will help the medical staff to better understand parents' needs. This understanding may be employed by developing strategies that assist parents of infants in NICU to cope. One way to enhance parents coping is by establishing a new program of parents' support group. Parents with previous positive experiences can be invited to share their experiences with parents of babies in the NICU. Another way is to raise medical-staff awareness about the parental need for physical, spiritual, and emotional support during their infant’s stay in NICU. In this regard, it is recommended that the medical staff assign a specific time to sit with the family and answer their questions and their worries regarding the evolvement of their infant’s case and provide guidance for after-discharge care whenever needed. More suggestions such as recruiting social workers to help parents in coping, encouraging the engagement of husbands and extended family members, and finding safe delivery mechanisms for the delivery of expressed breast milk from home to the NICU.
